# Simultaneous removal of endotoxins, inflammatory mediators and uremic toxins in ICU patients with septic shock: a retrospective cohort study

**DOI:** 10.1038/s41598-024-70522-3

**Published:** 2024-08-23

**Authors:** Benjamin E. Theisen, Christoph Lichtenstern, Christian Nusshag, Benjamin Tan, Tobias Hölle, Markus A. Weigand, Armin Kalenka, Mascha O. Fiedler-Kalenka

**Affiliations:** 1 Medical Faculty Heidelberg, Department of Anesthesiology, Heidelberg University, Im Neuenheimer Feld 420, 69120 Heidelberg, Germany; 2 Medical Faculty Heidelberg, Department of Nephrology, Heidelberg University, Im Neuenheimer Feld 162, 69120 Heidelberg, Germany; 3 Medical Faculty Heidelberg, Department of Pediatrics, Heidelberg University, Im Neuenheimer Feld 420, 69120 Heidelberg, Germany; 4 Kreiskrankenhaus Bergstraße, Viernheimer Str. 2, 64646 Heppenheim, Germany

**Keywords:** Sepsis, Septic shock, Renal replacement therapy, Inflammatory mediators, Endotoxins, Hemoadsorption, Cardiovascular diseases, Immunological disorders, Kidney diseases, Infectious diseases, Bacterial infection, Health care, Renal replacement therapy

## Abstract

Sepsis, one of the leading causes of death, is still lacking specific treatment. OXIRIS (BAXTER, Deerfield, IL, USA) is the first device allowing combined removal of endotoxins, inflammatory mediators and uremic toxins, alongside fluid balance control. Available data is very limited. This retrospective propensity score-matched cohort study of adult patients with septic shock aimed to evaluate septic shock duration and mortality in patients treated with either standard of care renal replacement therapy (RRT) or RRT with combined hemoadsorption, who were admitted to the interdisciplinary surgical intensive care unit at Heidelberg University Hospital during the years 2018 through 2021. Main outcomes were duration of shock, thirty-day mortality and plasma interleukin-6 levels before and after initiation of hemoadsorption. Included were 117 patients (female, 33%; male 67%); median age: 67 (16) years. After matching: 42 patients (female, 33%; male, 67%); mean age: 59.1 ± 13.8 years. There was no statistically significant difference in septic shock duration (*p* = 0.94; hazard ratio (HR) 0.97 (95% CI, 0.48–1.97)). Thirty-day survival analysis showed a non-statistically significant survival difference. (*p* = 0.063; HR 0.43 (95% CI, 0.17–1.09)). A post-hoc 90-day survival analysis revealed statistically significant longer survival and lower death hazard ratio in patients treated with RRT + HA (*p* = 0.037; HR = 0.42 (95% CI, 0.18–0.99). In conclusion, RRT with combined hemoadsorption of endotoxins, inflammatory mediators and uremic toxins is a modality worth further investigation.

## Introduction

Sepsis is one of the leading causes of death and is defined as a life-threatening organ dysfunction, caused by a dysregulated (immune) response of an organism to an infection^1^. Blood purification devices have been examined for sepsis therapy, capable of removing or inactivating cytokines and endotoxins^2^. Inflammatory molecules and cytokines relevant to the pathogenesis of sepsis are removed using a sorbent material in a filter. Several commercial applications are available, e.g., OXIRIS (BAXTER, Deerfield, IL, USA), CYTOSORB (CYTOSORBENTS Corp., Monmouth Junction, NJ, USA), and Polymixin-B (TORAYMYXIN, TORAY INDUSTRIES, Inc., Tokyo, Japan) – another commercially available material investigated in the past with controversial results.^3–5^.

Multi organ dysfunction syndrome (MODS) may be the result of disparities between pro- and anti-inflammatory cytokines and the ensuing so-called „cytokine-storm “^5,6^. Septic patients may develop immunosuppressive states, where suppression of the primary infection is unsuccessful and where superinfections may gain ground.^7^ Since the immune response of an organism relies on the secretion of mediators like cytokines, it seems reasonable to attempt the removal of these mediators to alleviate septic shock^5^.

Classic techniques of continuous veno-venous hemodialysis (CVVHD), continuous veno-venous hemofiltration (CVVHF) or continuous veno-venous hemodiafiltration (CVVHDF) are based on convection and diffusion; a relevant removal of inflammatory mediators or endotoxins from the blood has not been observed^5^. More recently, materials have been developed allowing the removal of said substances^5,8^. During the ongoing COVID-19 pandemic, an association between severity of disease and the dysregulated immune response has been observed^9,10^, which in turn led to increased interest in above-mentioned methods^5^.

Most of the available hemoadsorptive membranes focus on a single target, e.g., cytokine or endotoxin removal^11^. The OXIRIS filter is a hemoadsorptive device which can be used in combination with a hemofiltration device and is available for use in patients with sepsis and acute kidney injury (AKI) in some European and Asian countries^11^. Its highly adsorptive membrane is singular since it allows the combination of several properties, i.e., renal replacement therapy (RRT), removal of endogenous mediators (like cytokines) and exogenous molecules (like endotoxins), and presents anti-thrombogenic properties ^2,11^. It can be used with standard CRRT modalities^2^. In addition to RRT, the singular combination of the highly permeable (negatively charged) *AN69* membrane in combination with the (positively charged) PEI treatment in the OXIRIS filter achieves removal of both (positively charged) cytokines and (negatively charged) endotoxins respectively, and comes with built-in anticoagulation ^11–13^. It is currently among those hemoadsorptive devices with the highest adsorptive capabilities for endotoxins and cytokines, uniting an evacuation of cytokines (like CYTOSORB) and endotoxins (like TORAYMYXIN)^11,14^. Its application has been advocated in hemodynamically unstable septic shock-patients with or without AKI, considering favorable effects of the filter on endotoxin and cytokine levels in vitro, but opinions differ^15^. It is expected that patients with AKI are most likely to profit from its application^15^. Some authors conclude that treatment should be discontinued if unsuccessful in stabilizing hemodynamics within 72 h^15^.

### Interleukin 6 (IL-6)

The capability of reduction of IL-6 and other cytokines by treatment with hemoadsorption and comparable hemoadsorptive filters has been observed in vivo by previous authors^16–20^.

IL-6, secreted by T-cells and a stimulator of antibody secretion by B-cells, plays an important role in the so-called “cytokine storm” present in sepsis^16^. IL-6, IL-10 and TNF-alpha are among the mediators principally augmented during this process^21^. Increasing levels of IL-6 were found to be linked with increased mortality, while lower levels were related with recovery in septic shock^22^.

Data on hemoadsorption in septic shock is limited, especially for OXIRIS^11,23^. In animals, hemodynamic improvements and lower mortality have been shown, yet findings in humans have been ambiguous^11,24^. Data in humans, showing lower mortality, shorter duration of septic shock and lower rates of organ dysfunction, are mainly derived from two small single-center randomized trials with a number of 16 patients each^18,25^, small single-center retrospective studies, and case reports^2,12,13,16,18–20,25–34^.

There is no recommendation for routine treatment of septic shock patients with a hemoadsorptive device^35,36^, and there is a necessity for studies to further elucidate these applications^35^.

### Objectives

The aims of this study were to collect data about patients with renal replacement therapy (RRT) ± hemoadsorption (HA) using OXIRIS at our institution and to perform a retrospective explorative analysis.

The main study goal was defined as the duration to resolution of septic shock, as defined by the Sepsis-3 criteria, i.e.: need of vasopressors to achieve a mean arterial pressure (MAP) of ≥ 65 mmHg, and serum-lactate > 2 mmol/L (18 mg/dL)^1^. For the purpose of this study, the time to resolution of septic shock was defined as the time from onset of septic shock until a reduced vasopressor need had been achieved for ≥ 6 h (in terms of a norepinephrine-dosage of < 0.2 µg/kg/min lean body weight (Boer) ^37^), as well as serum-lactate < 2 mmol/L (18 mg/dL). This was considered as a resolution of refractory septic shock.

Secondary goals were 30 day-mortality since onset of septic shock and plasma cytokine levels of interleukin-6 (IL-6).

## Methods

### Study design

This is a retrospective single-center cohort study with propensity-score matching. Results are reported following the STROBE guidelines^38^.

### Setting

All patients treated at the interdisciplinary surgical intensive care unit at Heidelberg University Hospital between January 1st 2018 and December 31st 2021 were screened (end of follow-up: August 14th 2022). A local database research was done for patients with diagnosis of septic shock^1^ and RRT.

### Ethics approval and consent to participate

This study was approved by the ethics commission of the Medical Faculty of Heidelberg University (Heidelberg, Baden-Württemberg, Germany) under the number S-238/2022 and was performed in accordance with the Declaration of Helsinki. Informed consent was waived by the ethics committee for this retrospective study.

### Participants

Inclusion criteria were: age ≥ 18 years, septic shock as defined by Sepsis-3 criteria^1^.

Exclusion criteria were: patients not having been treated with RRT during the septic shock period, potentially confounding procedures like CYTOSORB therapy, Molecular Adsorbent Recirculation System (MARS, BAXTER, Deerfield, IL, USA), plasmapheresis, CO_2_ elimination using PRISMAX (BAXTER, Deerfield, IL, USA), history of heparin-induced thrombocytopenia (HIT) and heparin allergy (i.e., contraindication for OXIRIS) ^11^, or insufficient data.

### Data sources

Local electronic patient charts (COPRA, COPRA System GmbH, Berlin, Germany; LAURIS, NEXUS SWISSLAB GmbH, Berlin, Germany and SAP, Walldorf, Germany) were reviewed and data collection was done using MICROSOFT EXCEL (MICROSOFT, Redmond, WA, USA). Subsequent analysis was done using IBM SPSS STATISTICS 28.0. (IBM, Armonk, NY, USA).

### Data collection

The following data were collected: Age at time of onset of septic shock, sex, height, weight, body mass index (BMI), lean body weight (BOER), infection focus, prior medical conditions (history of cardiovascular disease [coronary heart disease, arterial hypertension, heart failure, cardiomyopathy, heart valve disease or vitium, arrhythmia, myocardial infarction, heart transplant], kidney failure, history of tumor disease, diabetes), history of smoking, history of alcohol consumption, allergy to heparin or HIT, Sepsis related organ failure (SOFA) scores, Acute Physiology and Chronic Health Evaluation (APACHE) scores, and Simplified Acute Physiology II (SAPS II) scores at time of admission to the ICU. Furthermore, the following were noted: times of onset of septic shock (earliest available documentation in medical records), treatment by RRT ± HA, other adsorptive treatments, initiation- and conclusion-times of RRT ± HA, most recent available plasma IL-6 levels within 24 h before initiation of HA and latest available levels after initiation of HA (up to 24 h of conclusion of HA), time of septic shock resolution (as defined), time of death, administration of other vasopressors than norepinephrine at either time of shock-resolution or death, time of last follow-up at our institution, suspected foci and blood culture results. Intervals were calculated between the times of interest. During data collection, times were noted with precision of up to 15 min-intervals. Times of death were noted as stated in medical records. In case patients had suffered multiple episodes of septic shock, the first such episode was considered, as well as the first-time employment of OXIRIS. Furthermore, data on the conditions for RRT and RRT + HA were collected and described.

### Statistics

The study was conceived as a monocentric, retrospective study. Statistics are reported following the SAMPL guidelines^39^.

#### Explorative analysis and evaluation of homogeneity

An explorative evaluation of data was performed. Descriptive statistics were generated for the overall study population, and differences between groups were compared to find possible differences. Normally distributed continuous variables are reported as mean ± standard deviation (SD); non-normally distributed variables as median (Interquartile Range, IQR)). In categorical variables, „n “-numbers and percentages are reported.

In continuous variables, normal distribution was verified using the Shapiro–Wilk test. Variables with normal distribution were tested using the t-test for independent variables, and the Mann–Whitney-U test was used in skewed data. Differences in proportions were verified using Fisher’s exact test considering the restricted patient numbers. Standardized mean differences (SMD) are reported for all variables. Small, medium, and large effect sizes were considered to be represented by SMDs of 0.2, 0.5, and 0.8, respectively^40,41^.

All reported tests are two-tailed and exact significance is reported. Significance levels were defined at α = 0.05. Since both groups were not homogenic across all baseline characteristics, a one-to-one (paired) propensity score matching was performed.

#### Missing data

Missing data are reported as “n (%)” and patients with missing data were excluded from tests.

#### Propensity score matching

Concerning the details of propensity score matching (PSM), we kindly refer to the literature^42–44^. Rosenbaum and Rubin^44^ defined the propensity score as the probability of allocation to a treatment dependent of observed baseline covariates. It summarizes all covariates into one variable describing the probability of having been subjected to the treatment ^45^. Distribution of covariates defining the propensity score is the same in treated and control groups for each value in the score. ^45^ Patients with an identical score have the same distribution of measured baseline covariates^42^. The estimated propensity score is usually calculated using a logistic regression model, regressing the treatment modality on monitored baseline covariates^42^.

Before matching, variables describing cardiovascular disease were recombined into a single dichotomous variable for presence or history of cardiovascular disease, defined as present if the patient had a history of any of the following: coronary heart disease, arterial hypertension, heart failure, cardiomyopathy, heart valve disease, arrhythmia, myocardial infarction, or history of heart transplant.

Patients were matched using: SOFA scores, age, history of cardiovascular disease, and history of tumor disease. These were chosen as a compromise of highly suspected influencing factors for treatment assertation of patients, and of the constraint of using a limited number of covariates given the restricted study population^45^. Variable selection was done without analyzing any outcomes^45^. Several matching model iterations were performed, and judged through balance and remaining sample size^46^. Assessment of the resulting model was done before proceeding to the analysis of outcomes^45^. Care was taken as to not lose any patients of the intervention group, considering low numbers. Best results (i.e., remaining sample size and appropriate balance^46^) were obtained using nearest neighbor caliper matching without replacement, with priority to exact matching, in random order, discussed by Austin et al.^43^ Caliper width was set to 0.15. Within the matched sample, comparisons of treatment effects may directly be made^42^.

As recommended by Austin et al.^42^, paired tests were used in the matched sample: paired t-tests for normally distributed continuous variables and the Wilcoxon-test for non-normally distributed continuous variables; McNemar’s test for differences in proportions in dichotomous variables.

#### Primary and secondary outcomes

##### Survival analysis and hazard ratios

For the Kaplan–Meier analysis^47^ regarding duration of septic shock, the time to event period was defined as the duration from onset of septic shock until resolution of refractory septic shock as defined earlier. Patients who died before resolution of refractory septic shock occurred were right-censored. Concerning survival analysis for 30 day-, respectively post-hoc 90 day-mortality, the time to event period was defined as the duration from onset of septic shock to death. Patients alive after 30, respectively 90 days, or who were lost to follow-up within the 30-/90-day period were right-censored. The assumptions of the Kaplan–Meier estimator were met^48^, i.e. one can expect censoring to be independent of the probability of developing the event of interest; furthermore, there is no reason for suspecting different survival probabilities at different times of the observation period. Survival curves were created for the RRT and RRT + HA groups. Results are reported as mean (SD) and median (IQR) with 95% confidence intervals (CI). Statistical significance was tested using the Log-Rank test (Mantel-Cox).

Univariate Cox regression^49^, as discussed by Austin^50^ was performed to estimate hazard ratios (HR). The time to event period was defined as the duration from onset of septic shock until resolution of refractory shock (primary outcome) or until death during the 30-/90-day follow-up after onset of septic shock (secondary outcome) and the treatment groups as binary predictor. HRs are reported with 95% CI.

##### Interleukin-6

To assess the evolution of IL-6 levels before and after initiation of HA, Wilcoxon’s signed-rank test was used for patients where IL-6 levels had been measured as described above. For patients with RRT without HA, regrettably IL-6 levels had not been measured during hospitalization and thus were not available.

## Results

### Population

**Fig. 1:**
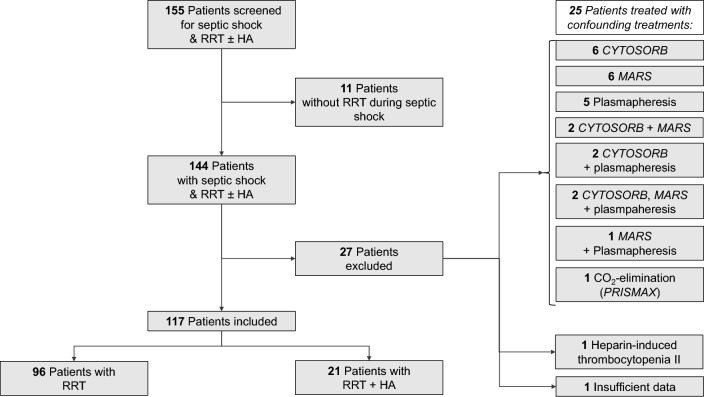
Flowchart showing the study inclusion process.

Screening identified 155 patients. Eleven patients were excluded since RRT was not applied during septic shock. Twenty-seven patients were excluded due to confounding treatments, HIT, or insufficient data. (Fig. 1). Eventually, 117 patients were included in the study.

### Descriptive data

Female patients represented 33%, male patients 67% of the study population. The median age was 67 (16) years at onset of septic shock. RRT + HA was performed in 21/117 patients (18%) and RRT in 96/117 patients (82%).

At time of ICU-admission, median scores were: SOFA, 14 (4); APACHE, 35 (9); SAPS II, 79 (22).

The study population is described in detail in Table 1.

**Table 1:** Baseline characteristics.

Variable		Study population (n = 117)					Matched cohort (n = 42)				
		RRT (n = 96, 82%)	RRT + HA (n = 21, 18%)	Total (n = 117, 100%)	SMD	*p* (two-tailed)	RRT (n = 21 (50%)	RRT + HA (n = 21, 50%)	Total (n = 42, 100%)	SMD	*p* (two-tailed)
Sex	Female	30 (31%)	8 (38%)	38 (33%)	0.145	0.61 ^a^	6 (29%)	8 (38%)	14 (33%)	0.177	0.69 ^d^
	Male	66 (69%)	13 (62%)	79 (67%)			15 (71%)	13 (62%)	28 (67%)		
Age (years)	NA	68.5 (16.0) [65.3–69.9]	58.0 (20.0) [52.4–64.0]	67.0 (16.0) [63.7–68.1]	**0.810**	**0.002** ^b^	60.1 ± 15.1 [53.2–67.0]	58.2 ± 12.7 [52.4–64.0]	59.1 ± 13.8 [54.8–63.5]	0.106	0.55 ^e^
Height (cm)	NA	174.1 ± 10.8 [172.0–176.3]	175.7 ± 10.1 [171.1–180.3]	174.4 ± 10.6 [172.5–176.3]	0.145	0.55 ^c^	177.6 ± 12.3 [172.0–183.2]	175.7 ± 10.1 [171.1–180.3]	176.6 ± 11.1 [173.2–180.1]	0.041	0.59 ^e^
Weight (kg)	NA	85.0 (29.8) [82.7–92.5]	85.0 (32.5) [79.1–104.7]	85.0 (29.5) [83.8–92.9]	0.173	0.66 ^b^	80.0 (31) [70.5–92.4]	85.0 (32.5) [79.1–104.7]	82.5 (36.0) [78.4–94.9]	0.452	0.10 f.
BMI (kg/m^2^)	NA	27.6 (9.0) [27.4–30.6]	26.6 (11.0) [25.7–33.6]	27.5 (9.0) [27.6–30.6]	0.087	0.90 ^b^	24.2 (8.8) [22.4–29.3]	26.6 (11.0) [25.7–33.6]	25.6 (7.8) [25.2–30.4]	0.445	0.09 f.
Smoker	Non-smoker	57 (60%)	15 (71%)	72 (61%)	0.124	0.80 ^a^	14 (67%)	15 (71%)	29 (69%)	0.085	> 0.99 ^d^
	Ever smoker	30 (31%)	6 (29%)	36 (31%)			5 (24%)	6 (29%)	11 (26%)		
	unknown	9 (9%)	0 (0%)	9 (8%)			2 (9%)	0 (0%)	2 (5%)		
Alcohol abuse	No abuse	71 (74%)	19 (91%)	90 (77%)	0.116	> 0.99 ^a^	15 (71%)	19 (91%)	34 (80%)	0.071	> 0.99 ^d^
	Abuse	11 (11%)	2 (9%)	13 (11%)			2 (10%)	2 (9%)	4 (10%)		
	unknown	14 (15%)	0 (0%)	14 (12%)			4 (19%)	0 (0%)	4 (10%)		
Coronary heart disease	no	61 (63%)	17 (81%)	78 (67%)	0.370	0.20 ^a^	15 (71%)	17 (81%)	32 (76%)	0.177	0.69 ^d^
	Yes	35 (37%)	4 (19%)	39 (33%)			6 (29%)	4 (19%)	10 (24%)		
Arterial hypertension	No	31 (32%)	9 (43%)	40 (34%)	0.266	0.31 ^a^	12 (57%)	9 (43%)	21 (50%)	0.156	0.73 ^d^
	Yes	65 (68%)	11 (52%)	76 (65%)			9 (43%)	11 (52%)	20 (48%)		
	unknown	0 (0%)	1 (5%)	1 (1%)			0 (0%)	1 (5%)	1 (2%)		
Heart failure	No	84 (88%)	18 (86%)	102 (87%)	0.053	0.73 ^a^	19 (91%)	18 (86%)	37 (88%)	0.253	> 0.99 ^d^
	Yes	12 (12%)	3 (14%)	15 (13%)			2 (9%)	3 (14%)	5 (12%)		
Cardiomyopathy	No	89 (93%)	19 (91%)	108 (92%)	0.083	0.66 ^a^	18 (86%)	19 (91%)	37 (88%)	0.124	> 0.99 ^d^
	Yes	7 (7%)	2 (9%)	9 (8%)			3 (14%)	2 (9%)	5 (12%)		
Heart defects (vitium)	None, or corrected	86 (90%)	19 (91%)	105 (90%)	0.029	> 0.99 ^a^	17 (81%)	19 (91%)	36 (86%)	0.177	0.69 ^d^
	Non-corrected pathological	10 (10%)	2 (9%)	12 (10%)			4 (19%)	2 (9%)	6 (14%)		
Arrhythmia	No	75 (78%)	18 (86%)	93 (80%)	0.187	0.56 ^a^	18 (86%)	18 (86%)	36 (86%)	0.177	> 0.99 ^d^
	Yes (atrial fibrillation)	21 (22%)	3 (14%)	24 (20%)			3 (14%)	3 (14%)	6 (14%)		
Past myocardial infarction	No	88 (92%)	21 (100%)	109 (93%)	0.330	0.35 ^a^	19 (91%)	21 (100%)	40 (95%)	0.317	0.50 ^d^
	Yes	8 (8%)	0 (0%)	8 (7%)			2 (9%)	0 (0%)	2 (5%)		
Past cardiac transplant	No	96 (100%)	19 (91%)	115 (98%)	**0.759**	**0.03** ^a^	21 (100%)	19 (91%)	40 (95%)	0.317	0.50 ^d^
	Yes	0 (0%)	2 (9%)	2 (2%)			0 (0%)	2 (9%)	2 (5%)		
Renal insufficiency	Acute	59 (61%)	17 (81%)	76 (65%)	0.410	0.13 ^a^	16 (76%)	17 (81%)	33 (79%)	0.071	> 0.99 ^d^
	Chronic (incl. acute-on-chronic)	37 (39%)	4 (19%)	41 (35%)			5 (24%)	4 (19%)	9 (21%)		
History of tumor disease	No	51 (53%)	18 (86%)	69 (59%)	**0.679**	**0.007** ^a^	15 (71%)	18 (86%)	33 (79%)	0.299	0.38 ^d^
	Yes	45 (47%)	3 (14%)	48 (41%)			6 (29%)	3 (14%)	9 (21%)		
Diabetes	No	64 (67%)	16 (76%)	80 (68%)	0.204	0.45 ^a^	11 (52%)	16 (76%)	27 (64%)	0.340	0.23 ^d^
	Yes (any type)	32 (33%)	5 (24%)	37 (32%)			10 (48%)	5 (24%)	15 (36%)		
SOFA (admission to ICU)	NA	14.0 (4.0) [12.5–13.8]	13.0 (5.0) [11.3–14.2]	14.0 (4.0) [12.5–13.6]	0.110	0.61 ^b^	12.5 ± 3.5 [10.9–14.1]	12.8 ± 3.1 [11.3–14.2]	12.6 ± 3.3 [11.6–13.6]	0.110	0.77 ^e^
APACHE (admission to ICU)	NA	36.0 (9.0) [33.5–36.3]	34.0 (9.0) [30.0–35.8]	35.0 (9.0) [33.3–35.8]	0.293	0.15 ^b^	32.3 ± 8.0 [28.7–36.0]	32.9 ± 6.4 [30.0–35.8]	32.6 ± 7.2 [30.4–34.9]	0.250	0.80 ^e^
SAPS II (admission to ICU)	NA	81.5 (23.0) [74.8–81.3]	72.0 (15.0) [66.5–79.1]	79.0 (22.0) [74.2–80,0]	0.334	0.11 ^b^	69.5 ± 17.2 [61.7–77.3]	72.8 ± 13.8 [66.5–79.1]	71.1 ± [66.3–76.0]	0.151	0.44 ^e^

Results are reported as n (%), mean ± SD, or median (IQR) with [95% CI]. Normal/non-normal distribution was verified using Shapiro-Wilk’s test, and the following tests used as appropriate – a, Fisher’s exact test; b, Mann-Whitney-U-Test; c, t-test (independent samples); d, McNemar’s test; e, Paired t-test; f, Wilcoxon signed-rank test. Exact significance is reported. SMD, standardized mean difference.

Medium effect sizes (i.e., SMD > 0.5) and *p*-values < 0.05 are shown boldunderline.

#### Comparison of both cohorts prior to matching

Both study cohorts were mostly homogenic among collected baseline characteristics. Substantial differences were seen in age (median age in years: RRT, 68.5 (16.0); RRT + HA 58.0 (20.0)), history of tumor disease (RRT, 45 (47%); RRT + HA, 3 (14%)) and cardiac transplant (RRT, none; RRT + HA, 2 (10%)).

The remaining variables were mostly balanced between groups (Table 1). Noticeable differences were observed in history of coronary heart disease (RRT, 35 (37%); RRT + HA, 4 (19%)), past myocardial infarction (RRT, 8 (8%); RRT + HA, none), and renal insufficiency.

### Propensity score matching

Propensity score matching (PSM) resulted in a collective of 42 patients. The model allowed all patients treated with HA to be matched.

**Fig. 2:**
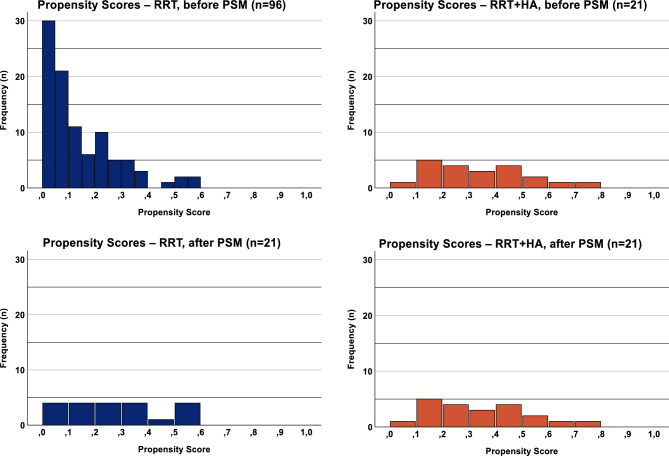
Distribution of Propensity Scores. Histograms showing the distribution of propensity scores before and after propensity score matching, demonstrating an appropriate adjustment of both study groups’ propensity scores.

Median propensity scores before matching: RRT, 0.10 (0.18); RRT + HA, 0.32 (0.33). After matching: RRT, 0.27 (0.28); RRT + HA 0.32 (0.33) (Fig. 2). Minimum, 0.01, maximum, 0.71. The matched sample consisted of 42 patients (female, 33%; male, 67%) with a mean age of 59.1 ± 13.8 years. Baseline characteristics were adequately balanced through PSM, however, there were some remaining differences notably in weight/BMI, coronary heart disease (RRT, n = 6; RRT + HA, n = 4), past myocardial infarction (RRT, n = 2; RRT + HA, none), past cardiac transplant (RRT, n = 0; RRT + HA, n = 2), diabetes (RRT, n = 10; RRT + HA, n = 5 and history of tumor disease (RRT, n = 6; RRT + HA, n = 3), which need to be kept in mind considering the limited patient population. (Table 1).

#### Treatment initiation

Several patients were already treated with some form of RRT before the onset of septic shock (RRT, n = 9/21; RRT + HA, n = 3/21). For patients with standard of care RRT in whom RRT was only initiated after the onset of septic shock (n = 12/21), the median duration from onset of shock to initiation of RRT was 39.3 (34.4) hours. Conversely, patients with RRT + HA in whom RRT was only initiated after the onset of septic shock (n = 18/21), the median interval to RRT initiation was 23.8 (21.7) hours, while the median interval from onset of septic shock to initiation of HA therapy was 24.5 (24.4) hours.

#### Description of RRT procedures

##### Membranes

RRT with GENIUS 90 was performed as sustained low-efficiency dialysis (SLED/CVVHD) (FRESENIUS MEDICAL CARE, Bad Homburg v. d. Höhe, Hesse, Germany) with FX 50 filters (FRESENIUS MEDICAL CARE, Bad Homburg v. d. Höhe, Hesse, Germany), with an effective surface area of 1.0 m^2^ and a fiber internal diameter (wet) of 185 µm. The FX 50 membrane is a polysulfone-based membrane called HELIXONE (FRESENIUS MEDICAL CARE, Bad Homburg v.d. Höhe, Hesse, Germany).

RRT with PRISMAFLEX was performed with PRISMAFLEX M150 sets (BAXTER, Deerfield, IL, USA) with an effective surface area of 1.5 m^2^ and a fiber wall thickness of 50 µm and a fiber internal diameter (wet) of 240 µm. PRISMAFLEX M150 sets include an AN 69 HF hollow fiber (Acrylonitrile and sodium methallyl sulfonate copolymer).

RRT ± HA was performed as CVVHDF using the PRISMAFLEX system (BAXTER, Deerfield, IL, USA). The OXIRIS hollow fiber consists of an acrylonitrile and sodium methallyl sulfonate copolymer and polyethyleneImine (surface treatment agent) and is heparin grafted (4500 ± 1500 IU/m^2^) and has been described in detail above.

##### RRT modality among cohorts

During septic shock, most patients with standard of care RRT were treated with SLED/CVVHD (n = 19/21; 90%), one patient (5%) with SLED/CVVHD as well as with CVVHDF (M150 filter) and one patient (5%) with CVVHDF (M150) alone. Net treatment durations during septic shock per modality are shown in Table 2.

**Table 2:** Treatment data for RRT during septic shock.

	RRT (n = 21)	RRT + HA (n = 21)
Hours treated with RRT ± HA during septic shock:		
SLED/CVVHD (FX 50)	12.9 (7.3–25.7) (n = 20)	9.0 (3.7–20.4) (n = 10)
CVVHDF (M150)	43.8 (40.3–47.3) (n = 2)	42.8 (0) (n = 1)
CVVHDF (OXIRIS)	NA	35.5 (16.1–51.5) (n = 21)

Results are reported as median (IQR). All patients with RRT+HA received CVVHDF with the OXIRIS filter during septic shock. Some patients received either therapy using SLED/CVVHD (FX 50) or CVVHDF (M150) during septic shock before switching to another modality or CVVHDF with OXIRIS. Therefore, the number of patients exceeds 100% of the total patient count.

Due to the retrospective nature of the study, patients unfortunately were not all treated using the same RRT modality. However, both treatment cohorts were relatively comparable in the duration of treatment during septic shock.

### Description of SLED/CVVHD

SLED/CVVHD are usually performed with blood flows around 100–150 mL/min with ratio of 1:1 or 2:1 to dialysate flow.

#### Anticoagulation

In SLED, anticoagulation was usually performed by a continuous infusion of unfractioned heparin, with target PTT levels of 40–60 s, adjustable depending of possible contraindications from the surgeons’ or intensivists’ point of view. In this patient cohort, patients received a median minimum heparin dose of 125 (125–423) IU/h and a median maximum heparin dose of 425 (125–545) IU/h during RRT and septic shock.

##### Dialysate

In SLED, dialysate fluids are chosen to match the patient’s sodium levels. Available solutions have concentrations of 135, 138, 140 or 145 mmol/L of sodium (Na^+^) and standardized bicarbonate levels of 35 mmol/L, which are only adjusted in case of severe acidosis or CO_2_ retention. Calcium (Ca^2+^) concentration is 1.5 mmol/L. Potassium (K^+^) concentrations are chosen according to the patient’s serum potassium levels, available solutions have concentrations of K^+^ of 2 mmol/L, 3 mmol/L, or 4 mmol/L, respectively.

#### Description of CVVHDF

CVVHDF was guided by an internal standardized protocol using PRISMAFLEX with a PRISMAFLEX M150 filter (both: BAXTER, Deerfield, IL, USA). Target blood flow rates are 100–180 mL/min citrate doses 2.0–4.5 mmol/L, initial calcium correction at 80%, dialysate rate 250–2000 mL/h, substitution fluid rate 200–1500 mL/h and Magnesium [10%] solution infused at 1 mL/h. Calcium levels are monitored extra-corporally 10 min after start of therapy and after each infusion rate change. Patient blood gases are taken 30 min after start of therapy and one hour after any change of flow rates, additionally to daily blood tests. Calcium is managed to keep extra-corporal levels of 0.25–0.35 mmol/L (standardized adjustment of citrate dose) and patient plasma levels of 1.1–1.3 mmol/L (adjustment of automated Ca^2+^-compensation rate + /− 10%). Base excess (BE) levels are targeted at − 2.5 to + 2.5 mmol/L and bicarbonate levels at 22–26 mmol/L. Anticoagulation is performed either by continuous infusion of heparin, or by locoregional anticoagulation with a citrate solution (PRISMOCITRATE 18/0, BAXTER, Deerfield, IL, USA) according to an internal standardized protocol. The citrate solution is composed as follows, and was infused in predilution mode: [Citrate, 18 mmol/L; Sodium (Na^+^), 140 mmol/L; Chloride (Cl^−^), 86 mmol/L].

PHOXILIUM (BAXTER, Deerfield, IL, USA) was used as substitution fluid in postdilution mode and as dialysate, usually with a therapeutic dose of 30–35 ml/kg/h. The composition is as follows: [Calcium (Ca^2+^), 1.25 mmol/L; Magnesium (Mg^2+^), 0.600 mmol/L; Sodium (Na^+^), 140.0 mmol/L; Chloride (Cl^-^), 115.9 mmol/L; Hydrogen phosphate (HPO_4_^2-^), 1.20 mmol/L; Hydrogen carbonate (HCO_3_^-^), 30.0 mmol/L; Potassium (K^+^), 4.00 mmol/L].

#### Infection focus

The majority of infection foci was abdominal (n = 26/42), followed by pulmonary (n = 4/42), and the combination of both (n = 4/42). Complete findings are reported in Table 3.

**Table 3:** Suspected foci and blood culture results.

Focus		Frequency (n)		
		Total	RRT	RRT + HA
Abdominal		26	15	11
Pulmonary		4	1	3
Combined pulmonary & abdominal		4	2	2
Abscess (muscular)		1	NA	1
Urologic		1	NA	1
Vasculary surgical		1	1	NA
Wound infection		1	NA	1
Combined pulmonary & central line		1	NA	1
Combined abdominal & central line		1	1	NA
Combined pulmonary, abdominal & central line		1	NA	1
*Unknown*		1	1	NA
Total		42	21	21
Blood Culture Result		Frequency (n)		
		Total	RRT	RRT + HA
Negative blood cultures		14	6	8
Gram positive		8	5	3
Gram negative		7	5	2
Combined gram positive & gram negative		5	4	1
Positive viral serology		5	NA	5
Fungal		1	NA	1
Combined gram positive & viral		1	1	NA
Combined gram positive, gram negative & fungal		1	NA	1
Total		42	21	21

Results are reported in absolute numbers (n).

#### Blood cultures/Serology

Blood cultures returned negative in 14 of 42 cases. Most frequent findings were gram positive (n = 8/42) and gram negative (n = 7/42) bacteria in blood cultures, followed by the combination of both (n = 5/42) and positive virus serology (n = 5/42). Complete findings are reported in Table 3.

#### Primary outcome

**Fig. 3:**
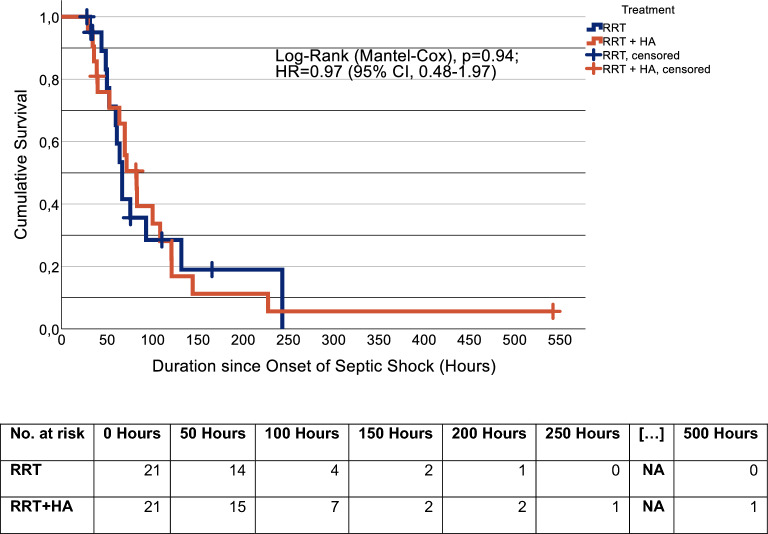
Duration of septic shock. Kaplan–Meier survival curves showing durations of septic shock for the matched cohort. The time to event period was defined as the duration from onset of septic shock until resolution of shock. Patients who died before resolution of refractory septic shock occurred were right-censored, and censored cases are marked with vertical bars. (Navy blue, renal replacement therapy (RRT); orange, renal replacement therapy with combined hemoadsorption (RRT + HA).

Septic shock resolution occurred in 32 of 42 patients (RRT, n = 14/21 (67%); RRT + HA, n = 18/21 (86%)) 10 of 42 patients died without resolution of refractory septic shock having occurred (RRT, n = 7/21 (33%); RRT + HA, n = 3/21 (14%); McNemar’s test (exact significance), *p* = 0.29). Overall for both cohorts, mean duration of shock was estimated at 110.9 h (95% CI, 70.6–151.1) with a median of 70.0 h (95% CI, 58.4–81.6). In patients with standard of care RRT, mean duration of shock was estimated at 102.3 h (95% CI, 64.3–140.4) with a median of 67.0 (95% CI, 59.4–74.6). For patients treated with RRT + HA, the estimated mean duration of shock was 111.6 (95% CI, 58.9–164.4) with a median of 83.0 h (95% CI, 56.2–109.8). The curves did not differ significantly one from another (Log-Rank (Mantel-Cox), *p* = 0.94). Univariate Cox-regression estimated the hazard ratio at 0.97 (95% CI, 0.48–1.97) for patients treated with RRT + HA. (Fig. 3).

At the time of refractory shock resolution and reduction of norepinephrine (as defined earlier), seven of 32 patients (22%) continued to receive additional vasopressor or inotropic therapy (dobutamine, 6/32 (19%)–four of which had a known history of cardiovascular disease; vasopressin, 1/32 (3%)).

#### Secondary outcomes

##### Kaplan–Meier estimator ^47^

**Fig. 4:**
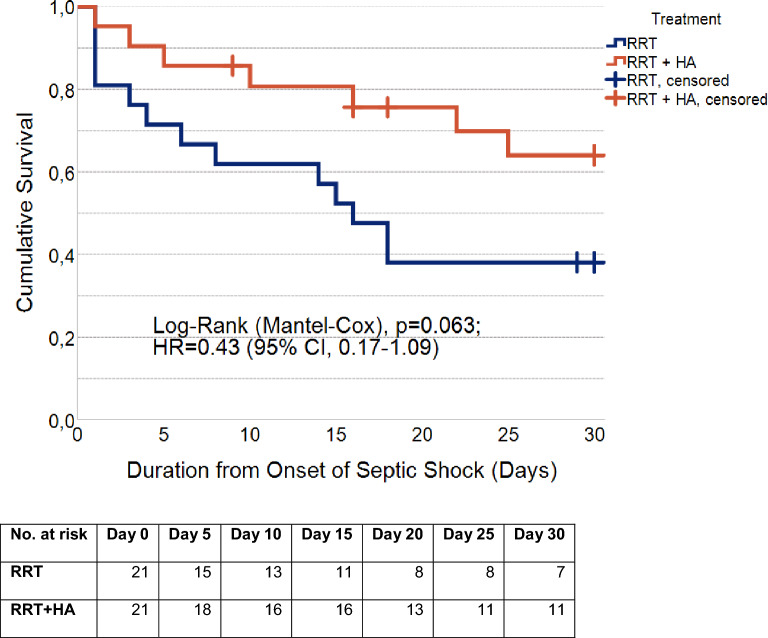
30-Day Survival. Kaplan–Meier survival curves showing 30-day survival for the matched cohort. Censored cases are marked with vertical bars. (Navy blue, renal replacement therapy (RRT); orange, renal replacement therapy with combined hemoadsorption (RRT + HA).

Within the 30-day follow-up period, 20 deaths were observed overall (RRT, n = 13; RRT + HA, n = 7). Overall, the mean survival time was estimated at 20.1 days (95% CI, 16.5–23.6). For patients with RRT, mean survival was estimated at 16.5 days (95% CI, 11.4–21.6) and median survival at 16.0 days (95% CI, 11.5–20.5), while for patients treated with RRT + HA, mean survival was slightly higher at 23.7 days (95% CI, 19.4–28.0). Median survival could not be computed for patients with RRT + HA, as more than half were alive after 30 days. The curves did not differ significantly (Log-Rank (Mantel-Cox), *p* = 0.063). Univariate Cox-regression estimated the hazard ratio (HR) at 0.43 for patients treated with RRT + HA (95% CI, 0.17–1.09). (Fig. 4).

**Fig. 5:**
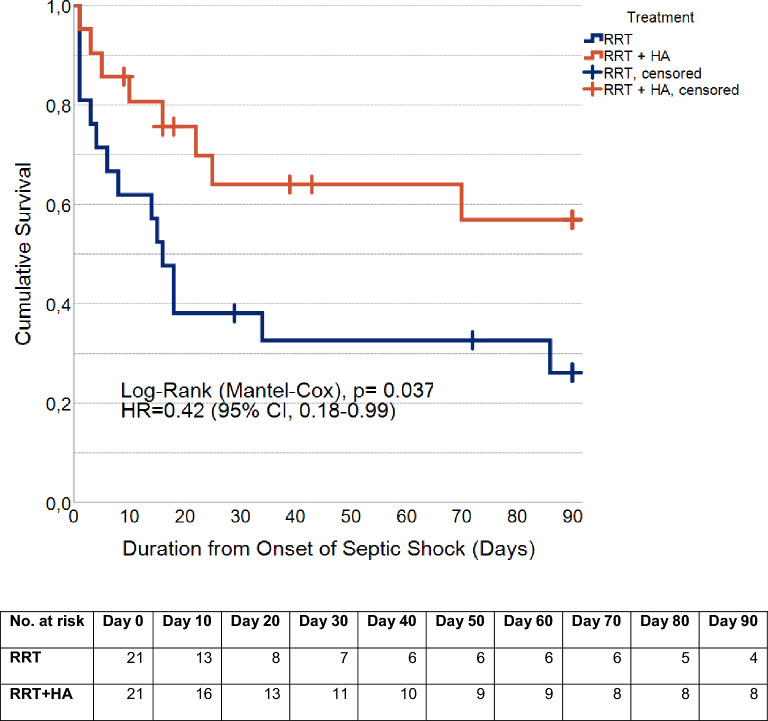
90-Day Survival. Kaplan–Meier survival curves showing 90-day survival for the matched cohort. Censored cases are marked with vertical bars. (Navy blue, renal replacement therapy (RRT); orange, renal replacement therapy with combined hemoadsorption (RRT + HA).

In a post-hoc 90-day survival analysis, 23 deaths were observed overall (RRT, n = 15; RRT + HA, n = 8). Overall, the mean survival time was estimated at 48.3 days (95% CI, 36.1–60.5). For patients with RRT, mean survival was estimated at 36.0 days (95% CI, 19.7–52.3) with a median of 16.0 days (95% CI, 11.5–20.5). For patients treated with RRT + HA, the mean survival time was estimated at 60.7 days (95% CI, 44.1–77.1) and median survival could not be computed as more than half of patients were still alive. The curves differed significantly one from another (Log-Rank (Mantel-Cox), *p* = 0.037). Univariate Cox-regression estimated the hazard ratio (HR) at 0.42 for patients treated with RRT + HA (95% CI, 0.18–0.99), corresponding to a 58% lower chance of death at any given time during the 90-day follow-up period. (Fig. 5).

##### Missing data for 30-day follow up

Explorative data analysis hinted at some differences in baseline characteristics of patients who were lost to follow-up at 30 days (n = 4/42) compared to patients with 30-day follow-up (n = 38/42), most notably they had a higher median age (71.0 (17.8) vs. 58.5 (19.3)) and higher median BMI (28.6 (22.5) vs. 25.6 (8.1)) Furthermore, the patients lost to follow-up did not have prior chronic kidney disease (n = 0/4 vs. n = 9/38) nor any history of tumor disease (n = 0/4 vs. n = 9/38), were more often known smokers (n = 2/4 vs. n = 9/38) and diabetics (n = 2/4 vs. n = 13/38).Prevalence of cardiovascular disease was comparable (n = 3/4 vs. n = 24/38), as was known alcohol abuse (n = 1/4 vs. n = 3/38)and there was a comparable number of females and males (females, n = 1/4 vs. n = 13/38). SOFA- (15.0 (9.3) vs. 13.0 (5.0)), APACHE- (34.0 (6.0) vs. 32.5 (11.3)) and SAPS II-scores (70.0 (38.3) vs.72.0 (20.3)) were comparable.

##### Missing data for 90-day follow-up

Patients lost to follow-up (n = 7/42) more often were female (n = 4/7 vs. n = 10/35) and older with a median age of 70.0 (18.0) vs. 58.0 (19.0). Body mass index in patients lost to follow-up were slightly lower with a median of 24.8 (9.5) vs. 26.4 (8.2), Rates of most other baseline characteristics, like cardiovascular disease (n = 5/7 vs. n = 22/35), known smokers (n = 2/7 vs. 9/35), alcohol abuse (n = 1/7 vs. 3/35), chronic kidney disease (n = 1/7 vs. n = 8/35) and history of tumor disease (n = 1/7 vs. n = 8/35) and diabetes (n = 4/7 vs. n = 11/35) and SOFA- (14.0 (9.0) vs. 13.0 (5.0)), APACHE- (34.0 (7.0) vs. 32.0 (11.0)) and SAPSII-scores (73.0 (26.0) vs. 72.0 (20.0)) were comparable.

#### Interleukin-6 levels

Information about interleukin-6 before and after hemoadsorption was available in ten patients and IL-6 levels were significantly reduced after initiation of hemoadsorptive therapy; (before HA: 1950 (12,824) pg/mL; after HA, 56 (210) pg/mL; *p* = 0.002; Wilcoxon signed-rank test).

## Discussion

### Key results

This retrospective, monocenter study showed no statistically significant differences in the primary outcome parameters duration of refractory septic shock nor thirty-day mortality. A post-hoc 90-day survival analysis showed statistically significant longer survival times and lower death hazard ratio for patients treated with RRT + HA. There was a statistically significant reduction of IL-6 levels after initiation of HA; however, there were no control measurements available.

### Generalizability

Due to the retrospective observational nature of this study, patients were not randomly assigned to treatments. Since in retrospective studies patients in treatment arms often diverge systematically from patients in control arms, propensity score matching was employed to balance treatment groups on observed baseline characteristics to attempt to balance systematic differences in both groups^42^. The association of patients with similar propensity scores allows for the replication of a quasi-randomized experiment^45^, imitating some of the characteristics of a randomized controlled trial^42^, and the effect of the treatment may then be compared directly between matched groups^42^. PSM may allow to produce a balance on measured baseline covariates, nonetheless, it is not possible to rule out heterogeneity in unmeasured covariates^42,51^.

#### Comparison to the literature

##### Mortality

Previous studies have found 90-day mortalities in septic shock of around 39%^52^, crude mortality of about 47%^53^, and 28-/30-day mortality of about 37%^54^, mentioning a substantial heterogeneity of mortality^52–54^. For example, Shankar-Hari et al. report a variation from 23% to about 82%^53^. Bauer et al.^52^ reported a median age of 64 years, comparable to this study’s population median age of around 59 years.

The overall 30-day mortality of around 48% (n = 20/42) and 90-day mortality of 55% (n = 23/42) in our matched study population is slightly higher compared to numbers reported in the literature^52–54^. The mean SOFA score at time of admission of 12.6 ± 3.3 with a median of 13.0 was substantially higher in our population compared to a median of 9.5 reported by Bauer et al. ^52^ Septic shock mortality rates were higher in retrospective (around 42%) than in prospective studies^52^. Per SOFA-score increase of one point, 90-day mortality increased by 2.4%^52^. An increase of 3.5 median SOFA score points (13.0 vs. 9.5) would translate into an expected 8.4% increase in mortality to about 47% (from around 39% reported by Bauer et al. ^52^.) for this study, approaching our findings. Patients in this study seem to have had higher morbidity considering higher SOFA scores and mortality.

Data on renal replacement therapy and hemoadsorption with OXIRIS is scarce. To the best of our knowledge, only two small randomized prospective studies^18,25^ and no studies with PSM have been published^23^. Most studies available on the subject report lower total patient populations. One recent work in 2022 reported comparable numbers, however, no adjustments were made in that case, making it difficult to compare outcomes from our point of view^16^. Interestingly, a recent systematic review and meta-analysis in 2023 found lower seven-, 14- and 28-day mortalities and shorter length of stay in the ICU, but in contrast to the present findings there was no statistically significant difference in 90-day mortality^55^. This meta-analysis however was criticized, among other things, because results from randomized controlled trials and retrospective studies were pooled and because most of the studies lacked detailed reporting^56^. Moreover, half of the considered studies did not report 28-day mortalities and the probable presence of publication bias was commented^57^.

##### Duration to therapy initiation

The median duration from onset of septic shock to initiation of HA therapy of 24.50 (24.38) hours corresponds well to previously described times of 18–46 h ^2,16,19,26^. While the duration to initiation of RRT + HA may seem rather long, this might be due to the fact that treatment with RRT + HA is administered only in select patients, and there is no standard procedure regarding its initiation. One reasonable explanation might be the duration until treatment is approved by a senior clinician. However, one needs to be mindful of these timespans as intervening to remove endotoxins and inflammatory mediators may make the most sense in the very first hours of septic shock. There has to be a careful assessment of benefits and risks for each patient, as there are risks involved with hemoadsorptive procedures and for some devices even negative outcomes have been reported^58^.

### Limitations

The number of patients with RRT + HA without exclusion criteria was limited. Regrettably, IL-6 levels were only available for patients with RRT + HA, since these were not measured for patients in the control group during hospitalization. Although both patient cohorts were adequately adjusted through the matching procedure, some differences remained. While less substantial than before matching, this has to be taken into consideration when interpreting the results, as in a small patient cohort even minor differences in characteristics such as past myocardial infarction might interfere with outcomes such as survival, and since patients lost to follow-up were somewhat older.

The fact that patients in the RRT and RRT + HA cohort were not all treated with the same modality or anticoagulants is obviously a clear limitation to this retrospective study. On the other hand, the limitation is relativized due to the lack of data supporting the superiority of any treatment modality in patients with acute kidney injury. Two relatively recent meta-analyses of studies comparing continuous RRT and SLED found no difference in recuperation of kidney function nor mortality^59,60^. A relatively recent meta-analysis on anticoagulants in continuous RRT concluded that there was no evidence for overall superiority of any anticoagulant. It was noted that citrate may have a benefit concerning major bleedings, but that there probably is no meaningful advantage regarding mortality after 28 days nor in preventing formation of clots^61^.

Furthermore, PSM may only adjust for measured baseline covariates, while unmeasured characteristics may remain unbalanced between groups^42^ and PSM may not stand in for randomized trials. Nevertheless, as has to be dealt with low patient numbers and regarding possible outcome improvements in sepsis, retrospective data must be taken into consideration.

### Interpretation

There was no statistically significant difference in the primary and secondary outcomes, i.e., duration of septic shock nor 30-day mortality, although patients in the RRT + HA cohort seemed to have a slight survival advantage. A post-hoc 90-day survival analysis revealed statistically significant longer survival and lower mortality for patients treated with RRT + HA. Considering some residual differences in baseline characteristics as described above, one has to be careful when interpreting these results as some of those characteristics might interfere with survival in patients treated with RRT + HA. Nevertheless, the most difference in survival probability seems to exist during the first 30 days, as this is where most patients died in the RRT cohort. Factors such as history of tumor disease might reduce overall survival probability, though this should impact survival in the medium rather than the short term. Factors such as coronary heart disease or past myocardial infarction however may interfere with short-term survival. It is conceivable that treatment with RRT + HA during the early phase of septic shock, attempting at preventing the development of immunosuppressive states and empowering the host organism to fight the primary infection may offer some survival benefit for a certain patient collective. The statistical non-significance of the present results during the first 30 days may result from the restricted patient cohort, on the other hand, the remaining discrepancies in baseline characteristics may have caused bias in survival analysis.

It would be more compelling to assume that a shorter duration of shock would be easier to demonstrate than lower mortality. However, there are other factors that might come into play, for example the severity of septic shock, or the different phenotypes of septic shock that were described in previous studies^62,63^. There may be undiscovered heterogeneity in therapeutic effects depending on the sepsis phenotype, however, specific analysis of these differences may not be recognized in many studies due to small sample sizes^62^, as is the case for the present investigation. This incoherence of treatment effects and clinical outcomes may also have had an influence on the differences in mortality but not in duration of septic shock seen in this investigation. Future studies on HA should keep in mind the existence of different sepsis phenotypes.

IL-6 levels were decreased in exploratory analysis after initiation of hemoadsorptive therapy, however as IL-6 was not measured in controls an interpretation of IL-6 levels is not possible.

It is currently unclear which patients are most likely to profit from hemoadsorption, although it is conceivable that both patients with gram-negative (with endotoxins) as well as with gram-positive bacterial infection (with translocation of gram-negative species from the digestive tract) may profit from its employment^11^. The use of hemoadsorption may be justified in certain patients; however, there were no statistically significant differences in the primary outcome parameters in this retrospective single center study. This study’s patient collective stems from a limited patient population at a large university hospital. Even so, compared to mostly still lower numbers reported in previous studies, the present results provide a considerable contribution to the available literature.

## Conclusion

In this retrospective monocenter cohort study, the primary and secondary outcomes of duration of septic shock and 30-day mortality were not statistically significantly different between patients treated with standard of care RRT and patients treated with RRT and hemoadsorption, although patients with hemoadsorption seemed to have a slight survival advantage.

Prospective randomized multicenter studies are warranted to further elucidate the role of simultaneous removal of endotoxins, inflammatory mediators and uremic toxins in ICU patients with septic shock, while care should be taken to account for different sepsis phenotypes.

## Data Availability

The data that support the findings of this study are not openly available due to reasons of sensitivity and de-identified data can be made available from the corresponding author upon reasonable request. A data use agreement and ethics commission approval will be sought as needed.

## Abbreviations

MODS: Multi organ dysfunction syndrome
CVVHD: Continuous veno-venous hemodialysis
CVVHF: Continuous veno-venous hemofiltration
CVVHDF: Continuous veno-venous hemodiafiltration
AKI: Acute kidney injury
RRT: Renal replacement therapy
HA: Hemoadsorption
MAP: Mean arterial pressure
IL-6: Interleukin-6
BMI: Body mass index
IQR: Interquartile range
PSM: Propensity score matching
SOFA: Sepsis-related organ failure assessment
APACHE: Acute physiology and chronic health evaluation
SPAS: Simplified acute physiology score
CI: Confidence interval
SD: Standard deviation
HIT: Heparin induced thrombocytopenia
